# Value-based and benefit-based strategies in deciding to bring a test into use should be distinguished

**DOI:** 10.1186/s41512-016-0003-9

**Published:** 2017-02-08

**Authors:** Werner Vach

**Affiliations:** Institute of Medical Biometry and Medical Statistics, Faculty of Medicine and Medical Center—University of Freiburg, Stefan Meier Str. 26, Freiburg, 79104 Germany

**Keywords:** Benefit, Biomarker, Diagnostic test, Enrichment designs, Interaction designs

## Abstract

**Background:**

Regulatory and health technology assessment agencies have commented differently on the question whether results from enrichment studies can be used to justify to bring a test into use. We try to provide a framework to discuss this issue.

**Results:**

Mathematical definitions for the value and the benefit of a new diagnostic test are given. The possible conclusions about value and benefit from enrichment studies and interaction studies are explored. The terms benefit-based strategy and value-based strategy are introduced. Several potential consequences of using one of the two strategies in deciding to bring a test into use are identified and quantified. Interaction designs allow to assess benefit and value. Enrichment designs allow only to assess benefit. However, it is often probable that interaction studies allow no firm conclusions about the value. The advantage of a benefit-based strategy stems mainly from allowing test-positive patients earlier or even ever to benefit. The main disadvantage is a potential delay in detecting tests of no value.

**Conclusions:**

Benefit-based strategies are preferable if the risk of off-label use and of delayed decisions on the value of a test can be limited. Otherwise, the superiority depends highly on research practice.

## Background

In developing new diagnostic tests and assessing their benefit for patients, enrichment designs are one popular design. In enrichment designs, only the test-positive patients are randomized to the two treatments of interest, typically the standard treatment currently given to all patients and a new treatment expected to improve patient outcomes in test-positive patients. Consequently, we can at the best only conclude that the new treatment is beneficial for test-positive patients. It may happen that other studies randomizing (also) test-negative patients demonstrate (later) that also the test-negative patients benefit from the new treatment. Then, there is actually no need for the test: We can improve patient outcomes just by giving the new treatment to all patients. In other words, the test is only of value, if it is justified to withhold treatment in test-negative patients [1].

This potential constellation raises the question about adequate conclusions about a new diagnostic test when only results from an enrichment study are available. Several regulatory or health technology assessment agencies already commented on this question. The FDA [2] expressed the wish that “…ideally …there will be at least some data on the marker negative population…”. The EMA [3] expressed its concerns by stating that “The regulatory acceptability of excluding…negative patients from trials will depend on the strength of evidence (plausibility, scientific rationale and clinical data) provides for the lack of effect in these patients.” The German IQWiG [4] simply concludes about enrichment designs that “…such designs allow conclusions only if other information allows to exclude that an effect observed in the randomized patients would also be present in the non-randomized patients^1^.” The FDA already pointed out a potential undesirable consequence of such a rigorous approach: “When the treatment is a critical advance for the enriched group, it would generally be unreasonable to delay approval for the enriched group, even if few data on the group without the enrichment factor were available …”.

To clarify the impact of such differences in the interpretation of results from enrichment designs, we contrast in this paper the benefit of a new test—i.e., the improvement in outcome—with the value of the new test—i.e., whether the test can be used to justify different treatment decisions in two groups of patients. We first introduce formal definitions of these two concepts. Next, we discuss conclusions we can draw about the value or the benefit of a new diagnostic test from results of randomized diagnostic studies following one of the three most common designs: interaction studies, enrichment studies, and comparative studies. Then, we consider two general strategies to make decisions about bringing a test into use, reflecting the abovementioned differences in the interpretation. We distinguish between value-based and benefit-based strategies and present several issues we have to take into account if we want to identify the better strategy.

## Methods

In the following, we assume that a diagnostic test *T* is applied in the patient population of interest. The test yields a positive test result with probability ${p^{T}_{+}}$ and a negative test result with probability ${p^{T}_{-}} := 1 - {p^{T}_{+}}$. The test should inform the choice between two interventions, which we denote by *i*_+_ and *i*_−_. We expect that test-positive patients benefit from *i*_+_ compared to *i*_−_, but that this does not hold for the test-negative patients. If we randomize patients to *i*_+_ or *i*_−_, we would observe in test-positive patients the treatment effect ${\theta ^{T}_{+}} := E(Y|T \! = \! +,I \! = \! {i_{+}}) - E(Y| T \! = \! +,I \! = \! {i_{-}})$ and in test-negative patients the treatment effect ${\theta ^{T}_{-}} := E(Y|T \! = \! -,I \! = \! {i_{+}}) - E(Y| T \! = \! -,I \! = \! {i_{-}})$. The random variable *Y* denotes here a patient-relevant outcome with large values representing a more favorable outcome. It may be measured on a binary, continuous, count, or time to event scale. We further assume that *i*_+_ represents an experimental treatment in the sense that we would prefer to choose *i*_−_ instead of *i*_+_ when we are forced to make a uniform choice for all patients.

In discussing the value of a diagnostic test, we are comparing three patient management strategies: giving *i*_−_ to both test-positive patients and test-negative patients (as we would do if the diagnostic test is not available), giving *i*_+_ to test-positive patients and *i*_−_ to test-negative patients, and giving *i*_+_ to both test-positive and test-negative patients. Each strategy implies a corresponding level of the average outcome:

$$\begin{array}{@{}rcl@{}} {e_{--}} &:=& E(Y | I = {i_{-}}, T = +) P(T = +)\\ && +\, E(Y | I = {i_{-}}, T = -)P(T = -) = E(Y| I = {i_{-}}) \\ {e^{T}_{+-}} &:=& E(Y | I = {i_{+}}, T = +) P(T = +) \\ &&+\, E(Y | I = {i_{-}}, T = -)P(T = -) \\ {e_{++}} &:=& E(Y | I = {i_{+}}, T = +) P(T = +)\\ &&+\, E(Y | I = {i_{+}}, T = -)P(T = -) = E(Y| I = {i_{+}}) \end{array} $$

The difference between ${e^{T}_{+-}}$ and *e*_−−_ reflects the benefit we expect from applying the test: We hope to be able to improve the average outcome by giving the test-positive patients the better treatment *i*_+_. Indeed, we have for this benefit *b* ^*T*^ the simple relation

$$\begin{array}{@{}rcl@{}} {b^{T}} &:=& {e^{T}_{+-}} - {e_{--}} = E(Y | I = {i_{+}}, T = +) P(T = +)\\ && +\, E(Y | I = {i_{-}},T = -) P(T = -) - E(Y| I = {i_{-}}) \\ &=& E(Y | I = {i_{+}}, T = +) P(T = +)\\ &&+ \,E(Y | I = {i_{-}},T = -)P(T = -)\\ && - \,(E(Y | I = {i_{-}}, T = +) P(T = +)\\ && +\, E(Y | I = {i_{-}},T = -)P(T = -)) \\ & =& E(Y | I = {i_{+}}, T = +) P(T = +)\\ && -\, E(Y | I = {i_{-}}, T = +)P(T = +) \\ &=& {\theta^{T}_{+}} {p^{T}_{+}} \end{array} $$

i.e., the benefit is positive if ${\theta ^{T}_{+}}$ is positive.

However, we cannot exclude that it is actually best to give *i*_+_ to all patients, i.e., that *e*_++_ is also larger than ${e^{T}_{+-}}$. This is actually the case if ${\theta ^{T}_{-}}$ is positive, as we have the simple relation ${e_{++}} - {e^{T}_{+-}} = {\theta ^{T}_{-}} {p^{T}_{-}}$. Hence, the test is of no value if both ${\theta ^{T}_{-}}$ and ${\theta ^{T}_{+}}$ are positive (or both are negative), as then it is optimal to give *i*_+_ to all patients (or *i*_−_ to all patients, respectively), and we cannot improve the average outcome by applying the diagnostic test. Only if ${{\theta ^{T}_{-}} \leq 0}$ and ${{\theta ^{T}_{+}} > 0}$ the test is of value, as its application allows us to achieve the optimal average outcome ${e^{T}_{+-}}$.

If there exists already a diagnostic test *T*_1_ to inform the choice between *i*_+_ and *i*_−_, and if we want to demonstrate that it is useful to replace *T*_1_ by *T*_2_, we have to show that we further improve the average outcome by using *T*_2_ instead of *T*_1_, i.e., that *e*+−*T*_2_>*e*+−*T*_1_. By the definition of *b* ^*T*^, this is equivalent to ${b^{T_{2}}} > {b^{T_{1}}}\phantom {\dot {i}\!}$. Hence, we have to demonstrate that *T*_2_ implies a larger benefit. However, this does not immediately imply that *T*_2_ is of value, as ${\theta ^{T_{2}}_{-}}>0$ is still possible. Only if *T*_1_ is of value, then we can conclude that *T*_2_ is of value if it improves the benefit. This follows from

$${} {e_{++}} - {e_{--}} = \left({e_{++}} - {e^{T}_{+-}}\right) + \left({e^{T}_{+-}} - {e_{--}}\right) = {\theta^{T}_{+}} {p^{T}_{+}} + {\theta^{T}_{-}} {p^{T}_{-}} $$

So, if we improve the benefit when using *T*_2_ instead of *T*_1_, we increase ${\theta ^{T}_{+}} {p^{T}_{+}}$, and hence, we decrease ${\theta ^{T}_{-}} {p^{T}_{-}}$, as the sum remains constant. Consequently, if ${\theta ^{T_{1}}_{-}}$ is non-positive, ${\theta ^{T_{2}}_{-}}$ must be non-positive, too.

Our definition of the benefit coincides with that given for the benefit from a new biomarker by different authors [5–8]. It should be distinguished from concepts like decision curve analysis and net benefit [9, 10]. These concepts rely on the availability of a reference standard and describe certain aspects of the accuracy of a test. However, knowledge about ${\theta ^{T}_{+}}$ and ${\theta ^{T}_{-}}$ may be useful to define the weights for false-negative or false-positive decisions required within such concepts. For the case of paired diagnostic studies, this idea has been exemplified in [11].

## Results

### Enrichment designs

In studies following an enrichment design [12] (also called targeted or selection design), we first apply the diagnostic test *T* in all patients in the population of interest. Patients with a positive test result are then randomized to either *i*_+_ or *i*_−_, and the outcome *Y* is only measured in these patients. Consequently, in an enrichment design, we can estimate ${\theta ^{T}_{+}}$ from this subpopulation. To demonstrate that there is a benefit from the test, it suffices to demonstrate ${{\theta ^{T}_{+}} > 0}$. This can be approached by performing a standard hypothesis test on ${H_{0}: {\theta ^{T}_{+}} \leq 0}$.

Obviously, we cannot estimate ${\theta ^{T}_{-}}$ from an enrichment design because in test-negative patients, the outcome *Y* is not measured. Hence, in general, we cannot make any conclusion about the value of the test. Exceptions may occur, if it is impossible to apply the treatment *i*_+_ in test-negative patients, e.g., if the test is detecting lesions which have to be treated. A controversial point, however, will often be that the choice of an enrichment design is based on the expectation that the treatment *i*_+_ does not work in the test-negative patients due to the underlying biological or clinical model. However, such an expectation is no empirical proof.

### Interaction designs

In interaction designs, both test-positive as well as test-negative patients are randomized to *i*_+_ and *i*_−_. Consequently, we can estimate both ${\theta ^{T}_{+}}$ and ${\theta ^{T}_{-}}$, and we can perform corresponding inference. A benefit can be proven as above by demonstrating ${{\theta ^{T}_{+}} > 0}$. To demonstrate that the test has a value, we have in addition to demonstrate ${{\theta ^{T}_{-}} \leq 0}$. In analogy, we can approach this by performing a test of ${H_{0}: {\theta ^{T}_{-}} > 0}$. However, we are now confronted with the challenge that this test may have very low power: Even if the test *T* has no value, the true value of ${\theta ^{T}_{-}}$ may be close to or exactly 0. Consequently, we may be willing to accept that we cannot prove ${{\theta ^{T}_{-}} \leq 0}$, but only ${\theta ^{T}_{-}} \leq \theta _{M}$, where *θ*_*M*_ is an appropriately chosen margin. This margin should reflect that even if ${\theta ^{T}_{-}}$ is above 0, but still below *θ*_*M*_, we would prefer to use *i*_−_ instead of *i*_+_ in the test-negative patients, for example, because of safety concerns about *i*_+_. However, typically, this still implies that we need many more test-negative patients than test-positive patients to come to a decision about the value. To power the test for $H_{0}: {\theta ^{T}_{-}} > \theta _{M}$, we will consider ${\theta ^{T}_{-}}=0$ as the relevant alternative. To power the test for ${H_{0}: {\theta ^{T}_{+}} \leq 0}$, we will assume an effect *θ*_*A*_ in the test-positive patients which is typically distinctly larger than *θ*_*M*_. For example, if *θ*_*M*_ is one third of *θ*_*A*_, then we need approximately nine times more test-negative patients than test-positive patients to reach the same power. So, the feasibility of this approach is highly depending on whether we have a sufficient number of test-negative patients. The setting of the JUPITER study [13] may serve as an example where such an approach may be feasible [14].

Instead of trying to demonstrate that the test has a value on top of a proven benefit, we may alternatively try to demonstrate that the test has no value, as this would allow us to omit the test in the future. Here, we have to demonstrate ${\theta ^{T}_{-}}>0$, which we can approach by testing $H_{0}: {\theta ^{T}_{-}} \leq 0$. Again, we may be confronted with the challenge of limited power, as even if ${\theta ^{T}_{-}}$ is above 0, it will be often smaller than the effect in the test-positive patients. Moving to demonstrate ${\theta ^{T}_{-}} > \tilde \theta _{M}$ for some-negative margin $\tilde \theta _{M}$ is here not a convincing alternative as it is hard to justify to recommend *i*_+_ to test-negative patients if it is slightly worse than *i*_−_.

In reflecting about whether we have the power to make a statement about the value of the test, it might be useful to remember that most diagnostic tests are based on dichotomizing a continuous (bio-)marker *X* at some prespecified cut point *c*. Then, we can consider the treatment effect as a function of *X*, i.e.,

$$\theta(x) = E(Y | I \! = \! {i_{+}}, X=x) - E(Y | I \! = \! {i_{-}}, X=x) $$

and we have ${\theta ^{T}_{+}} = E (\theta (X) | X > c) $ and ${\theta ^{T}_{-}} = E(\theta (X) | X < c)$. We can now consider two specific situations, which may be often met in practice. In the first situation, *i*_+_ is defined by an add-on to *i*_−_, such that we expect that all patients still can benefit from *i*_−_ if *i*_+_ is chosen, and the test-positive patients may have an additional benefit by the add-on. So it is reasonable to assume that *θ*(*x*) is positive or at least above a negative value close to 0 in the majority of test-negative patients and only of substantial magnitude close to the cut point. Consequently, ${\theta ^{T}_{-}}$ is also close to 0, and we have to expect difficulties in proving that the test has no value as well as that it has a value. A second situation is given when *i*_+_ is a complete replacement of *i*_−_, so we cannot expect a benefit by giving *i*_+_ when *X* is small. This means we have *θ*(*x*)<0 for *x* below a cutpoint $\tilde {c}$, and *θ*(*x*) is distinctly below 0 for the majority of patients below this cutpoint. If *c* and $\tilde {c}$ roughly coincide, we have ${\theta ^{T}_{-}} < 0 $ and probably distinctly below 0, and hence, we have sufficient power to demonstrate that the test has a value. On the other side, we must be aware of that even if the test has a value, it might be still suboptimal in a case where *c* and $\tilde {c}$ differ substantially.

Two further remarks are necessary at this step. First, even if we ask both the question whether the test is of value (and accept the margin approach) and whether it is of no value, we may fail to come to a definite conclusion: Both the test on $H_{0}: {\theta ^{T}_{-}} > \theta _{M}$ and the test on $H_{0}:{\theta ^{T}_{-}} \leq 0 $ can be insignificant. Actually, this is what we have to expect for a diagnostic test *T* with $ {\theta ^{T}_{-}}$ between 0 and *θ*_*M*_ (and slightly above and below this range) as then both tests lack power. Second, our considerations about analyzing interaction studies are different from what you can find in the clinically oriented literature [15–17]. There the emphasis is on demonstrating ${{\theta ^{T}_{+}} > 0}$ in the first place and ${\theta ^{T}_{-}} >0 $ in the second place, but not in demonstrating ${\theta ^{T}_{-}} \leq 0$, as the latter has no implications on treatment choice. Furthermore, there is also interest in the overall treatment effect *θ*_all_=*e*_++_−*e*_−−_, in particular to demonstrate *θ*_all_>0, if the results allow to demonstrate ${{\theta ^{T}_{+}} > 0}$ and support ${{\theta ^{T}_{-}}>0}$ without allowing to demonstrate the latter. Consequently, studies following an interaction design may require a new statistical analysis independent of the analysis performed by the principal investigator, if we want to make a decision about the value of the test. As such an analysis involves up to three null hypotheses (${\theta ^{T}_{+}} \leq 0, {\theta ^{T}_{-}} > \theta _{M}, {\theta ^{T}_{-}} < 0$), an adequate handling of multiplicity has to be planned. If the results of the interaction study have been used to determine the cutpoint *c*, further statistical considerations are necessary [8].

Actually, there are two different variants of interaction designs. In randomize-all designs [18], all patients are randomized and the test is applied in all patients. In biomarker-stratified designs [19], the test is first applied in all patients, and then in either the test-positive or the test-negative patients, randomization is restricted to a subsample, typically aiming at similar number of patients in both groups. However, if the aim is to demonstrate the value of the test, our considerations above suggest to aim at more test-negative than test-positive patients in such designs. The design has also an influence on estimating ${p^{T}_{+}}$ in order to estimate the benefit. In the randomize all designs, we can just use the randomized patients, whereas in the biomarker stratified design, we have to make use of all patients, not only the randomized ones.

### Comparative randomized diagnostic studies

Comparative studies aim at comparing two diagnostic tests *T*_1_ and *T*_2_. In randomized comparative diagnostic studies, patients are randomized to either $\mathcal {T}=T_{1}$ or $\mathcal {T}=T_{2}$, and only the corresponding test is applied in each patient. The intervention *i*_+_ is applied in the case of a positive test result, and *i*_−_ is applied in the case of a negative test result. The outcome *Y* is measured in all patients. The two arms can then be compared by looking at the difference in the mean outcomes. This means we try to estimate the effect

$$\begin{array}{@{}rcl@{}} \tilde \theta &:=& E (Y| \mathcal{T}=T_{2}) - E(Y | \mathcal{T}=T_{1}) \\ & = &E(Y| I = {i_{+}},T_{2}=+) P(T_{2}=+)\\ && +\, E (Y | I = {i_{-}}, T_{2}=-)P(T_{2}=-) \\ && -\, (E (Y| I = {i_{+}},T_{1}=+) P(T_{1}=+)\\ && +\, E (Y | I = {i_{-}}, T_{1}=-)P(T_{1}=-)) \\ &=& {b^{T_{2}}} + E(Y|I = {i_{-}}) - ({b^{T_{1}}} + E(Y|I = {i_{-}})) \\ &=& {b^{T_{2}}} - {b^{T_{1}}} \end{array} $$

i.e., the additional benefit.

Note that in this design, we do not randomize the treatment. In test-positive patients, we always use *i*_+_, and in test-negative patients, we always use *i*_−_. Hence, none of the quantities *θ*+*T*_1_,*θ*+*T*_2_, ${\theta ^{T_{1}}_{-}}$, and ${\theta ^{T_{2}}_{-}}$ are estimable. Consequently, we cannot assess the value of *T*_2_ or *T*_1_. However, as pointed out in the last section, if we demonstrate an additional benefit of *T*_2_ and we already know that *T*_1_ is of value, then we can conclude that *T*_2_ is of value, too.

### The basic dilemma

Each country has today established a regulatory system to control the process of bringing new drugs into use. This process may include marketing approval, accessibility in a publicly financed health care system, or decisions on reimbursement. In many countries, these systems are also responsible for corresponding decisions on new diagnostic tests. For drugs, decisions are typically based on a proof of an increased efficacy, i.e., improvements in outcomes, together with considerations about safety or other risks. Ignoring for a moment the latter and focusing on efficacy, with respect to a diagnostic test, we are confronted with the question whether we should require a proof of the value of the test or a proof of the benefit of the test.

There can be little doubt that from a societal perspective, it is desirable to bring only tests with a value into use. Using a test with no value implies that we deny patients with a negative test result to benefit from receiving *i*_+_ instead of *i*_−_ and that the society has to spend resources on applying the test *T*, although we can obtain even a larger benefit just by giving *i*_+_ to all patients. However, if the benefit of a test has been proven and we are still lacking a proof of its value, insisting on such a proof has a consequence which is not desirable: We deny patients with a positive test result to benefit from *i*_+_ despite of a proven benefit. This situation appears if a new diagnostic test with no current competitor has been already successfully investigated in an enrichment design, but not (yet) in an interaction design.

### Consequences of applying benefit-based and value-based strategies for the decision on a single test

If we want to understand the advantages and disadvantages of requiring a proof of the benefit or a proof of the value, it is necessary to regard such decisions as part of a general strategy applied to all new diagnostic tests: The benefit-based strategy (BBS) requiring a proof of the benefit and the value-based strategy (VBS) requiring a proof of the value (which automatically implies a proof of the benefit). In BBS, a test is brought into use when we can demonstrate a benefit based on a study following an enrichment design or a study following an interaction design, which failed to come to a decision on the value. We may later revise this decision in the sense to decide to provide *i*_+_ to all patients and to stop using the test, when we can demonstrate that the test is of no value. This can be achieved by a study following an interaction design or an randomized controlled trial (RCT) in test-negative patients only. In VBS, there is only one decision at the time point when the value or no value of the test has been demonstrated. In the first case, the test is brought into use and the test-positive patients receive *i*_+_, in the second case, *i*_+_ is just given to all patients. Such a decision will be typically based on a study with an interaction design, but it is also possible to base it on separate RCTs in test-positive and test-negative patients.

In Table 1, we illustrate some consequences of using BBS or VBS using artificial study results from the development of six different tests. We consider the case of one study following an enrichment design and/or one (later) study following an interaction design. For the first test, the enrichment study demonstrated ${\theta ^{T}_{+}} >0$ and in a later interaction study, it could be demonstrated that ${\theta ^{T}_{-}}$ is negative. Under BBS, the test would be in use after the enrichment study and under VBS after the interaction study. For the second test, the enrichment study demonstrated the benefit, but the interaction study later revealed that ${\theta ^{T}_{-}}$ is positive, i.e., the test is of no value. Under BBS, the test would be in use in the time period between the enrichment study and the interaction study, under VBS never. For the third test, the enrichment study demonstrated the benefit, but the interaction study remained inconclusive with respect to the sign of ${\theta ^{T}_{-}}$. Consequently, under BBS, the test would be used after the enrichment study, under VBS, it would be never used. For the fourth test, only an enrichment design study was performed, demonstrating ${\theta ^{T}_{+}}>0$. Under BBS, the test would be used after this study, under VBS, it would never be used. For the fifth test, only an interaction design study was performed, which demonstrated both ${\theta ^{T}_{+}}>0$ as well as ${\theta ^{T}_{-}}<0$. Under both strategies, the use of the test would start after the study. For the sixth test, we have again only an interaction study. This study demonstrated ${\theta ^{T}_{+}}>0$, but remained inconclusive with respect to ${\theta ^{T}_{-}}$. Under BBS, the test would come into use after the study, under VBS, it would never come into use.

**Table 1:** Some examples for the impact of the results of one enrichment design study and/or one interaction study on the time where the test is in use following BBS or VBS, respectively

Test number	Enrichment design study	Interaction design study		Time interval of test use under	
	${\hat \theta ^{T}_{+}}$	${\hat \theta ^{T}_{-}}$	${\hat \theta ^{T}_{+}}$	bbs	vbs
1	0.41 [0.11, 0.51] ^a^	–0.21 [–0.39, –0.03] ^a^	0.57 [0.39, 0.75] ^a^	E- *∞*	I- *∞*
2	0.54 [0.29, 0.78] ^a^	0.23 [0.01, 0.44] ^a^	0.39 [0.17, 0.52] ^a^	E-I	never
3	0.66 [0.34, 0.99] ^a^	0.11 [–0.21, 0.43]	0.47 [0.13, 0.82] ^a^	E- *∞*	never
4	0.29 [0.07, 0.51] ^a^	–		E- *∞*	never
5	–	–0.32 [–0.57, –0.06] ^a^	0.71 [0.56, 0.95] ^a^	I- *∞*	I- *∞*
6	–	–0.07 [–0.43, 0.57]	0.53 [0.07, 0.99] ^a^	I- *∞*	never

Treatment effects are expressed as log relative risks for treatment success with 95% confidence intervals.

^a^Log RR significant different from 0 at the 5% level. E and I refer to the time points of the enrichment and the interaction study

**Fig. 1:**
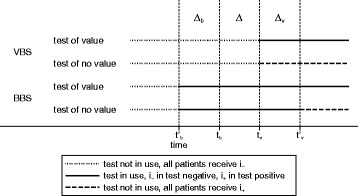
Relevant time points and durations when comparing BBS to VBS for a test with ${\theta ^{T}_{+}}>0$

We now start with considering more generally the consequences of following the two strategies on a single new test *T* with ${\theta ^{T}_{+}}>0$. Let *t*_*b*_ denote the time point where the benefit of the test is proven and *t*_*v*_ the time point where the value or no value of the test is proven, under the assumption of following a VBS. Let *Δ*:=*t*_*v*_−*t*_*b*_≥0 denote the duration where the test is not (yet) used in spite of having a proof for the benefit (cf. Fig. 1). In the case of following a BBS, we have to take into account that the time points of obtaining a proof of the benefit or a proof of the value or of no value may change. In particular, we may expect that a proof of the benefit is obtained earlier—as the test developers can bring the test into use by such a proof and hence are more eager to conduct an enrichment study early—and that a proof of the value is delayed—as the test developers have no interest to stop the use of the test and hence may hesitate to perform an interaction study or an RCT in test-negative patients. Hence, we denote by $t^{\prime }_{v}$ and $t^{\prime }_{b}$ the corresponding time points under BBS. With *Δ*_*b*_:=*t*_*b*_−*tb*′, we denote how much earlier we obtain a proof for a benefit, and with *Δ*_*v*_:=*tv*′−*t*_*v*_, we denote how much later we obtain a proof for a value. We do not require these numbers to be positive, although this is likely to be the case.

There are at least six different relevant consequences of the choice of the strategy, which are considered in Table 2 with respect to following BBS instead of VBS. First, by bringing a test with proven benefit into use, test-positive patients start to benefit from *i*_+_, independent of the value of the test. This starts in the case of BBS earlier by *Δ*+*Δ*_*b*_. Second, the same applies to any harm implied by exposure to *i*_+_, e.g., if there are safety issues. Third, as long as we do not have detected that a test has no value, test-negative patients will receive *i*_−_ and cannot benefit from *i*_+_. The time point of detecting and changing this is delayed by *Δ*_*v*_ in the case of a BBS. Fourth, there may be harm to patients by exposure to the diagnostic test. If the test has a value, this means that this harm starts earlier by *Δ*+*Δ*_*b*_; if the test has no value, this will happen in a period of length *Δ*+*Δ*_*b*_+*Δ*_*v*_ instead of never. The same argument applies to the costs implied by using the test—the fifth consequence considered in Table 2. The monetary costs of a test are of course independent of whether the test has a value or is of no value. However—as pointed out above—we may regard the costs as differing in quality, as in the case of a test with a value these costs are inevitable in order to improve patient outcome, whereas this is not the case for a test with no value. However, prior to *t*_*v*_ or $t^{\prime }_{v}$, respectively, we do not know that this may be the case, so we cannot criticize the use of these resources prior to these time points. A sixth relevant consequence has been pointed out by the FDA [2]: If a benefit is proven, but the value is still unknown, there may happen off-label use in the test-negative patients after approving *i*_+_ for test-positive patients. This off-label use hopefully stops once it is proven that the test has a value, i.e., that there is no clinically relevant treatment effect in the test negative patients. If the off-label use happens in all test-negative patients, then this decreases the average outcome if the test has a value, and it increases the average outcome if the test has no value. If the-off label use is selective—in particular, if the continuous marker *X* is taken into account and only patients with values close to the current cut point are selected for off label use—then the average outcome may increase even in the case of a test with no value.

**Table 2:** Six consequences of using BBS instead of VBS for a single new diagnostic test

		Test has a value	Test has no value
1)	Test-positive patients can benefit from *i*_+_	Starts earlier by *Δ*+*Δ*_*b*_	Starts earlier by *Δ*+*Δ*_*b*_
2)	Harm to test-positive patients by exposure to *i*_+_	Starts earlier by *Δ*+*Δ*_*b*_	Starts earlier by *Δ*+*Δ*_*b*_
3)	Test-negative patients can benefit from *i*_+_	Not applicable	Delayed by *Δ*_*v*_
4)	Harm to all patients by exposure to test	Starts earlier by *Δ*+*Δ*_*b*_	In a period of length *Δ*+*Δ*_*b*_+*Δ*_*v*_
5)	Use of resources for testing all patients	Starts earlier by *Δ*+*Δ*_*b*_	In a period of length *Δ*+*Δ*_*b*_+*Δ*_*v*_
6)	Additional off-label use	In a period of length *Δ*+*Δ*_*b*_+*Δ*_*v*_—harm to patients probable	In a period of length *Δ*+*Δ*_*b*_—harm to patients less probable

In summarizing the insights we can obtain from Table 2, let us start with looking at the first five consequences and assuming *Δ*_*v*_=0. Then, evaluating the consequences of using BBS instead of VBS for a single test involves only the consequences 1), 2), 4), and 5) and reduces to a comparison of the gain in efficacy when applying *i*_+_ instead of *i*_−_ in test-positive patients with the harms implied in these patients and with the harms and costs implied by applying the test in all patients, i.e., traditional benefit-risk and cost-benefit analyses. We have only to take in addition into account that this comparison concerns a limited time interval of length *Δ*+*Δ*_*b*_, and we have to remember that benefit, costs, and risk should refer to all patients, not only to test-positive ones. In particular, the benefit is given by *b* ^*T*^ and not by ${\theta ^{T}_{+}}$. Note also that these considerations are independent of whether the test is of value or is of no value. In contrast, consequence 6) differs between tests of value and tests of no value.

If *Δ*_*v*_>0, we have to take into account additional negative consequences in the case of a test with no value, as we prolong the period where test-negative patients do not benefit from *i*_+_, and we prolong the period of additional costs and potential harm by application of the test in all patients. In the case of a test with a value, we also prolong the period of a possible and probably harmful off-label use (Table 1).

### Further aspects in comparing BBS and VBS

In evaluating the advantages and disadvantages of using BBS or VBS, it is important to understand that *Δ* and *Δ*_*v*_ can be infinite. In the “Interaction designs” section, we have pointed out the difficulty to come to a decision about the value of a test when ${\theta ^{T}_{-}}$ is close to 0. So for such a test, it is likely that *Δ*=*∞* holds, as we will never be able to perform a study of sufficient size. Similarly, *Δ*=*∞* holds for all tests, where we hesitate to perform studies in test-negative patients, as the clinical or biological model suggests ${\theta ^{T}_{-}} \leq 0$. In any case, *Δ*=*∞* implies that only under BBS test-positive patients can ever benefit from getting *i*_+_.

The case *Δ*_*v*_=*∞* means that we never perform a study with an interaction design or an RCT in test-negative patients as consequence of using BBS instead of VBS. This may happen if such a study is in principle feasible, but after establishing the benefit in an enrichment study, there is no further incentive to perform such a study. In this case, the basic advantage of VBS is just that we ever detect that a test has no value and that test-negative patients then can benefit from *i*_+_.

The choice of BBS instead of VBS may, however, not only imply that we start to use enrichment designs earlier or instead of interaction studies or wait longer until we start interaction studies after performing enrichment studies. It might also imply that tests of no value, which would never been developed in the case of VBS, are now developed and never detected as being of no value.

Finally, it should be noted that the strategy chosen has also an impact on decisions about new diagnostic tests with an existing comparator. In BBS, we have to prove the additional benefit. In VBS, we have to require in addition that we know that the existing comparator is of value. In the long run, this constitutes no problem as the comparator could only come into use after proving its value. However, when introducing VBS as a new principle, we may lack such a proof for the comparator.

### Superiority of benefit-based or value-based strategy

Our considerations in the previous sections suggest that for a single test, BBS can imply a disadvantage compared to VBS if *Δ*_*v*_>0 (and in particular, if *Δ*_*v*_=*∞*) or/and if off-label use harms patients. This may be avoided by corresponding additional means, for example, a conditional approval requiring to perform studies allowing a decision on the value of the test within a certain time period and strict restrictions on off-label use. Then, for each test, patients will benefit from BBS compared to VBS, but at the societal level, there is still the risk for introducing more tests with no value. If such means are not available or it cannot be guaranteed that they work, it is more challenging to make statements about the superiority of one approach over the other, and further considerations are necessary. First, for a test with ${\theta ^{T}_{+}} \leq 0$ and ${\theta ^{T}_{-}} \leq 0$, we expect no difference, as under both strategies we should never start to use the test and all patients will always receive *i*_−_. If ${\theta ^{T}_{+}} > 0$ and ${\theta ^{T}_{-}} \leq 0$, i.e., the test is of value, BBS is of advantage. Averaging over all tests of value, the tests with *Δ*=*∞* make the dominating contribution, as then only under BBS patients will ever benefit. If ${\theta ^{T}_{-}}>0$ and ${\theta ^{T}_{+}}>0$, i.e., the test is of no value, VBS can be of advantage avoiding a delay in allowing test-negative patients to benefit from *i*_+_. Averaging over all tests of no value, the tests with *Δ*_*v*_=*∞* and *Δ*<*∞* make the dominating contribution, as then only under VBS test-negative patients will ever benefit. So roughly speaking, we have to find out whether there will be more tests of value with *Δ*=*∞* or more tests of no value with *Δ*_*v*_=*∞* and *Δ*<*∞*, if we assume that ${e^{T}_{+-}}-{e_{--}}$ and ${e_{++}}-{e^{T}_{+-}}$ are on average of the same magnitude in these two groups. These frequencies reflect the proportion of tests of value and of no value among all tests, the frequency of not performing interactions studies or performing interactions studies with insufficient power to demonstrate ${\theta ^{T}_{-}} \leq 0$ among tests of value, and the frequency of not performing interactions studies for tests of no value after successfully performing enrichment studies, even if this is feasible and would have been done if no enrichment study has been performed. Hence, the decision about the superiority of each strategy depends mainly on the research practice, and how a country can influence it in general and by the chosen strategy. A conditional approval mentioned above is one example for how to take a direct influence. In general, from a societal perspective, it is desirable that interaction studies are performed to sort out tests of no value. However, interaction studies require to randomize also the test-negative patients, even if there is little hope that any of these patients can benefit from *i*_+_, and—as *i*_+_ is typically a new therapy with unexplored safety profile—some likelihood for harm. Hence, societal interests may be in conflict with patient interests.

## Discussion

Our considerations demonstrate that it is useful and necessary to distinguish between the value and the benefit of a diagnostic test. The two perspectives have a direct impact on the analysis and interpretation of diagnostic studies, requiring the use of different approaches. Equaling value with benefit should be avoided.

Benefit and value are also fundamental corner stones in the strategies countries can choose to control the process of bringing diagnostic tests into use. The considerations of the IQWiG in its methodological guideline suggest that the IQWiG is favoring a value-based strategy. The considerations of the FDA suggest that the FDA can see some danger in such a strategy and that it favors a modified benefit-based strategy in the sense that even in the case of a proven benefit, they want to take a look at a rough estimate of ${\theta ^{T}_{-}}$. Such a modification does not change the considerations of this paper fundamentally, as also under such a strategy a test might be brought into use without having a proof for its value, and the benefit for the test-positive patients may be delayed, if we hesitate to bring it into use when we have a proof for this benefit. It will be of interest to see which options other countries will choose.

The question about superiority of a benefit-based or a value-based strategy cannot be answered in general. It depends on the research practice and the means a country has to influence the research practice. Interactions studies are desirable from a societal perspective, but not necessarily from a patient perspective.

Our considerations are only based on logical arguments and do not involve empirical data. Hence, the basic limitation of the paper may result from wrong arguments or from overlooking arguments. We could demonstrate that actually many different issues make a contribution to the discussion about the advantages and disadvantages of the two strategies. Hence, it is desirable to continue this discussion in order to identify wrong or overlooked arguments.

We would finally like to mention four further limitations:

1. We assume in our considerations that hypothesis tests never fail. The impact of the type I error on the strategies is not taken into account. In particular, we ignored that tests with ${\theta ^{T}_{+}} \leq 0$ may be erroneously regarded as having a benefit.
2. We also ignored the case ${\theta ^{T}_{-}}>0$ and ${\theta ^{T}_{+}}\leq 0$, i.e., tests with a value but an incorrect expectation about which patient group will benefit from the intervention.
3. We also ignored the possibility that during the development of a test, we may obtain evidence for *θ*_all_>0, e.g., from an RCT comparing *i*_+_ with *i*_−_ without applying the test.
4. We considered only single studies as evidence base. In reality, several studies may be available, and we have to use meta analytical techniques.

## Conclusions

The benefit and the value of a diagnostic test are two different concepts which need to be distinguished. Decisions to bring a test into use can be based on a proof of its benefit or a proof of its value. The superiority of these two strategies is highly depending on the research practice and its interaction with the strategies. Benefit-based strategies are preferable if the risk of off-label use and of delayed decisions on the value of a test can be limited.

## Endnote

^1^ Translation by the author.

## Abbreviations

BBS: Benefit-based strategy
EMA: European Medicines Agency
FDA: Food and Drug Administration
IQWiG: Institut für Qualität und Wirtschaftlichkeit im Gesundheitswesen
RCT: Randomized controlled trial
VBS: Value-based strategy
